# Cytokine gene expression assay as a diagnostic tool for detection of *Mycobacterium bovis* infection in warthogs (*Phacochoerus africanus*)

**DOI:** 10.1038/s41598-019-53045-0

**Published:** 2019-11-11

**Authors:** Eduard O. Roos, Leeré A. Scott, Sedzani Ndou, Francisco Olea-Popelka, Peter E. Buss, Lin-Mari de Klerk-Lorist, Robin M. Warren, Paul D. van Helden, Tashnica T. Sylvester, Michele A. Miller, Sven D. C. Parsons

**Affiliations:** 1 Department of Science and Technology/National Research Foundation Centre of Excellence for Biomedical Tuberculosis Research, South African Medical Research Council Centre for Tuberculosis Research, Division of Molecular Biology and Human Genetics, Faculty of Medicine and Health Sciences, Stellenbosch University, PO Box 241, Cape Town, 8000 South Africa; 2 Department of Clinical Sciences, College of Veterinary Medicine and Biomedical Sciences, Colorado State University, 300W. Drake Rd, Fort Collins, Colorado, 80523 USA; 3 Veterinary Wildlife Services, South African National Parks, Kruger National Park, Private Bag X402, Skukuza, 1350 South Africa; 4 Department of Agriculture, Forestry and Fisheries, Office of the State Veterinarian, Kruger National Park, PO Box 12, Skukuza, 1350 South Africa; 5 Present Address: The Pirbright Institute, Ash Rd, Pirbright, Woking, GU24 0NF United Kingdom

**Keywords:** Chemokines, Infectious diseases, Infectious-disease diagnostics, Diagnostic markers

## Abstract

*Mycobacterium bovis* infection has been described in many wildlife species across Africa. However, diagnostic tests are lacking for many of these, including warthogs (*Phacochoerus africanus*). Most literature on suids has focused on using serological tools, with few studies investigating the use of cell-mediated immune response (CMI) assays. A recent study showed that warthogs develop measurable CMI responses, which suggests that cytokine gene expression assays (GEAs) may be valuable for detecting *M*. *bovis*-infection, as shown in numerous African wildlife species. Therefore, the aim of the study was to develop GEAs capable of distinguishing between *M*. *bovis*-infected and uninfected warthogs. Whole blood was stimulated using the QuantiFERON-TB Gold (In-Tube) system, using ESAT-6 and CFP-10 peptides, before determining the relative gene expression of five reference (*B2M*, *H3F3A*, *LDHA*, *PPIA* and *YWHAZ*) and five target (*CXCL9*, *CXCL10*, *CXCL11*, *IFNG* and *TNFA*) genes through qPCR. The reference gene *H3F3A* was the most stably expressed, while all target genes were significantly upregulated in *M*. *bovis*-infected warthogs with the greatest upregulation observed for *CXCL10*. Consequently, the *CXCL10* GEA shows promise as an ante-mortem diagnostic tool for the detection of *M*. *bovis*-infected warthogs.

## Introduction

There are few validated ante-mortem diagnostic tests available to determine the *Mycobacterium bovis* infection status of warthogs (*Phacochoerus africanus*). Warthogs may serve as potential maintenance hosts of bovine tuberculosis (bTB) in *M*. *bovis*-endemic areas and the lack of appropriate diagnostic tools is an obstacle to understanding the dynamics of this disease^1,2^.

Serological assays have been used to distinguish between *M*. *bovis*-infected and uninfected warthogs^3^. However, cell-mediated immune responses (CMI) have been shown to have greater sensitivity in many species^4^. The CMI is activated early after mycobacterial infection and leads to the production of Th1 cytokines^4^. These cytokines play an important role in the initial control of the infection reducing progression of disease and bacilli multiplication^5^. Many CMI assays are based on the *in vitro* stimulation of whole blood (WB) to detect antigen-specific production of Th1 cytokines, such as interferon gamma (IFN-γ) and tumour necrosis factor alpha (TNF-α)^5,6^. While these types of CMI assays have been used to detect *M*. *bovis*-infected cattle and African buffalo, very few have investigated their use in suids^7^.

The QuantiFERON TB Gold (In-Tube) system (QFT), which makes use of the antigens early secretory antigenic target 6 kDa (ESAT-6) and culture filtrate protein 10 kDa (CFP-10), has been used to stimulate *M*. *bovis*-specific responses in whole blood (WB) from a variety of species^8–12^. They are more specific than the commonly used bovine purified protein derivative, which is a crude extract of *M*. *bovis*. These antigens have been shown to be highly immunogenic in a wide range of species^8–13^.

Although assays to detect antigen-specific CMI responses are commonly used in humans and livestock^7^, their utility is limited in wildlife by the lack of host-specific reagents^2^. However, based on the homology of cytokine sequences between related domestic and wildlife species, cytokine gene expression assays (GEAs) may provide diagnostic techniques for wildlife^9,10,14^. Candidate biomarkers such as *IFNG* and those in the *CXCL* family, which are induced by IFN-γ, have been investigated using gene expression assays (GEAs) in wildlife^9,10,14^.

The aim of this study was to optimise GEAs for the detection of *M*. *bovis* infection in warthogs, targeting *IFNG* and *TNFA*, as well as genes that are induced by IFN-γ, including *CXCL9*, *CXCL10* and *CXCL11*, which have been successfully used in other species to detect tuberculosis^9,10,14^. To achieve this, we developed relative quantitative real-time PCRs for transcripts of selected candidate reference and target genes of warthogs, identified the optimal reference gene (most stably expressed rgene), and calculated the relative target gene expression in response to ESAT-6 and CFP-10 in *M*. *bovis*-infected and uninfected warthogs.

## Results

The median amount of RNA extracted from each blood sample was 1 560 ng (range: 324–11 772 ng). The 260/280 and 260/230 ratios had a median of 1.97 (range: 1.53–2.77) and 0.64 (range: 0.05–2.14), respectively.

All reference and target gene sequences derived from warthogs showed the greatest identity to the domestic pig sequence, as compared to that of the cow (Fig. S1). Additionally, the qPCR primer binding sites had a high identity with the pig sequences (Table 1). The qPCR products had characteristic melt curves (Table 1) and were confirmed to be specific by sequencing (data not shown). Furthermore, no products were formed in the no-RT control qPCRs, nor were there products in the no-template controls for each qPCR. For all qPCRs, intra-assay variability was low (<1%) and amplification efficiencies ranged from 90 to 115% (Table 1).

**Table 1:** Primer sequences and assay parameters of quantitative polymerase chain reactions for selected gene transcripts of warthogs.

Gene	Primer sequence (5′-3′)	Reverse	T_annealing_	Pig identity	E%	AF	R^2^	IVA (%)	DM peak (°C)
	Forward								
*B2M*	CTCACTGTCTGGCCTGGATG	GGCGGATGGAACCCAGATAC	60 °C	F: 100%; R: 100%	103	2.03	0.99	0.5	82.5
*H3F3A*	AAACAGATCTGCGCTTCCAG	ACGTTTGGCATGGATAGCAC	60 °C	F: 100%; R: 100%	105	2.05	0.99	0.4	80.5
*LDHA*	TGCAACATGGCAGCCTTTTC	ACAACCAGCCTAGAGTTTGC	62 °C	F: 100%; R: 100%	111	2.11	0.99	0.5	79.5
*PPIA*	TGAGTGGTTGGATGGCAAAC	TGGTCTTGCCATTCCTGGAC	60 °C	F: 100%; R: 100%	114	2.14	0.99	0.7	77.5
*YWHAZ*	TTCTGAACTCCCCAGAGAAAGC	GCGTGCTGTCTTTGTATGACTC	62 °C	F: 100%; R: 100%	109	2.09	0.99	0.3	77.5
*CXCL9*	TCATCTTCCTGACTCTGCTTGG	TGGATCATCCTTTGGCTGGTG	62 °C	F: 95%; R: 100%	97	1.97	0.99	0.8	77.0
*CXCL10*	CCCACATGTTGAGATCATTGCC	TCTCTCTGTGTTCGAGGAGATC	60 °C	F: 77%; R: 100%	108	2.08	0.99	0.5	76.5
*CXCL11*	AAAGCGGGAAGGTGTCTTTG	GGCATCTTCGTCCTTTATGTGC	62 °C	F: 100%; R: 100%	105	2.05	0.99	0.7	78.0
*IFNG*	AGGCCATTCAAAGGAGCATG	AGTTCACTGATGGCTTTGCG	60 °C	F: 100%; R: 100%	115	2.15	0.98	1.0	78.5
*TNFA*	GGCCCAAGGACTCAGATCATC	ATACCCACTCTGCCATTGGAG	62 °C	F: 100%; R: 100%	101	2.01	0.98	1.0	81.5

E%: Amplification efficiency*;* AF: Amplification factor; IVA: Intra-assay variability; DM: Derivative melt curve peak temperature; F: Forward primer identity; R: Reverse primer identity.

Of the five reference genes, *H3F3A* was the most stably expressed, and was therefore chosen as the reference gene for further analyses (Fig. S2). The efficiencies of all target gene qPCRs were similar to the selected reference gene (data not shown).

**Figure 1:**
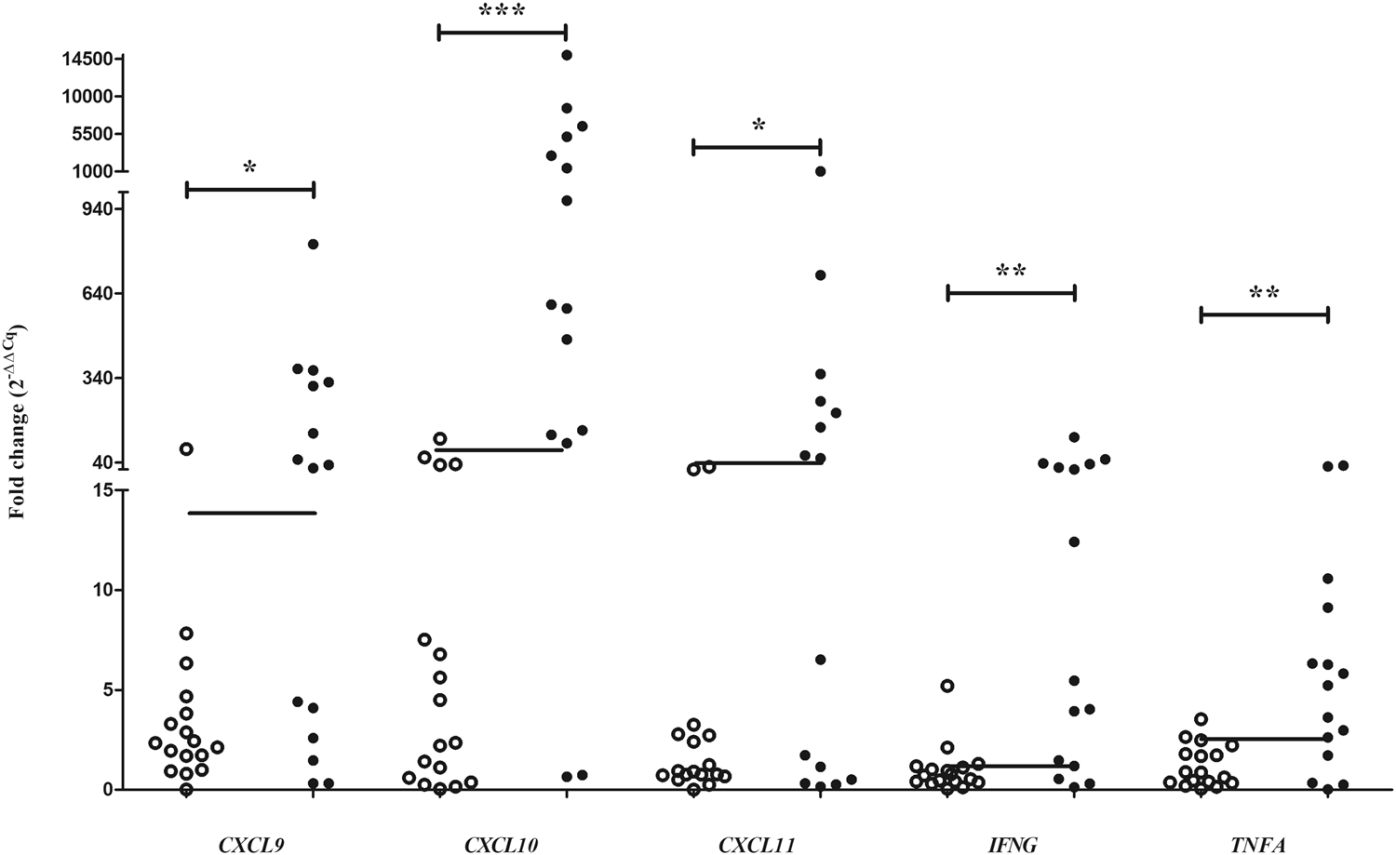
Antigen-specific expression of selected genes in response to ESAT-6/CFP-10 stimulation of blood from *M*. *bovis* uninfected (○) and *M*. *bovis*-infected (●) warthogs. Lines indicate gene specific diagnostic cut-off values. *p < 0.05, **p < 0.01, ***p < 0.001.

For *M*. *bovis*-infected warthogs, all target genes showed significant upregulation in response to antigen stimulation compared to uninfected animals (Fig. 1). Of these, *CXCL9*, *CXCL10*, and *CXCL11* showed the greatest upregulation, with median fold increases of 32, 601 and 55, respectively (Fig. 1). Of the target genes, *CXCL10* showed the greatest differential response between *M*. *bovis*-infected and uninfected warthogs (Fig. 1).

Cut-off values for all target genes were determined (Figs 1 and S3, Table 2). The sensitivity of *CXCL10* was the highest (87%; 95% CI: 60–98%), while *CXCL11* had the highest specificity (100%; 95% CI: 80–100%) (Table 2). Furthermore, *CXCL10* had the highest Youden’s index value of 0.81, thus making it the most accurate marker of the five target genes (Table 2).

**Table 2:** Test parameters of gene expression assays for the detection of *Mycobacterium bovis* infection in warthogs.

Target	Cut-off value	Se	95% CI	Sp	95% CI	J
*CXCL9*	>13.8	60	32–84%	94	71–100%	0.54
*CXCL10*	>83.5	87	60–98%	94	71–100%	0.81
*CXCL11*	>39.7	53	27–79%	100	80–100%	0.53
*INFG*	>1.18	80	52–96%	82	57–96%	0.62
*TNFA*	>2.55	73	45–92%	88	64–99%	0.61

Se: Sensitivity; CI: confidence interval; Sp: Specificity; J: Youden’s index value.

## Discussion

This study, describes the successful development of novel cytokine gene expression assays to distinguish between M. bovis-infected and uninfected warthogs. Among the five reference genes evaluated, *H3F3A* was the most stably expressed. Although all target transcripts (*CXCL9*, *CXCL10*, *CXCL11*, *IFNG* and *TNFA*) showed significant upregulation, demonstrating their utility as potential diagnostic markers of *M*. *bovis* infection, *CXCL10* was the most accurate and showed the greatest antigen-specific upregulation.

The mRNA coding regions and q-PCR primer binding sites of warthogs showed high identity with those of the domestic pig. This was expected since warthogs have common ancestry with domestic pigs and share many of their immune genes with other suids^15,16^. Consequently, the warthog GEA has potential to be used as another measure of CMI responses in other closely related suids.

The most stable reference gene was *H3F3A*, followed by *YWHAZ*. Previous studies for which *H3F3A* was reported to be a stable reference gene used other tissue types, such as PBMCs and alveolar macrophages, from *M*. *bovis*-infected cattle^17,18^. However, studies using WB identified *YWHAZ* as an appropriate reference gene in lion and hyena^8,9^. Nevertheless, *H3F3A* is a good reference candidate, as it is highly expressed in various tissue types^19^. Additionally, *H3F3A* and other genes in the H3.3 family are highly conserved across metazoans^19^, making this gene a candidate that might be used as a reference gene across a wide range of species.

For all five target genes, there was significantly greater antigen-induced expression in *M*. *bovis*-infected warthogs compared to uninfected animals. The *CXCL* genes showed greater antigen-induced expression levels in WB of *M*. *bovis*-infected warthogs than either *IFNG* or *TNFA*. The higher expression of the *CXCL* genes, compared to *IFNG*, have also been reported in lion and hyena^9,10^. This may be because *CXCL* genes are expressed by a greater number of cells (neutrophils, eosinophils, lymphocytes and monocytes) compared to *IFNG* (antigen stimulated Th1 cells) during antigen stimulation^20–26^. Studies have shown that chemokines and cytokines that are induced by IFN-γ can be used as sensitive and reliable biomarkers^26,27^. Therefore, the *CXCL* chemokines could be more suitable diagnostic candidates in GEAs.

Expression of *CXCL10* was the greatest among the targets as well as the most accurate marker for *M*. *bovis*-infection in warthogs. Previous studies have shown that *CXCL9* can be used as a diagnostic biomarker for *M*. *bovis*-infected lions, and that *CXCL11* is significantly up-regulated in *M*. *bovis* exposed hyenas^9,10^. This indicates the necessity to identify the most appropriate biomarker for the species being studied. Nevertheless, *CXCL10* may be a more suitable GEA biomarker for *M*. *bovis* infection in warthogs as a previous study identified IP-10, encoded by *CXCL10*, as a potential biomarker for *M*. *bovis* in warthogs^8^.

A limitation in this study was that the optimum incubation time for warthog WB was not determined prior to measuring the gene expression of each target gene^28,29^. In humans, peak mRNA levels for *IFNG* are detected after 4–6 h of incubation after which it returns to nearly background levels after 24 h^29,30^. This may be an additional explanation why *IFNG* had lower antigen-induced gene expression levels than *CXCL10* in *M*. *bovis*-infected warthogs. Another limitation was that we were restricted to only include warthogs from *M*. *bovis* endemic regions, as samples were collected opportunistically.

Future studies should focus on determining if GEAs could be used in other suid species, to identify *M*. *bovis*-infected individuals. Furthermore, the optimum antigen incubation time of WB should be determined in this species for both gene expression and protein production.

In summary, all five target genes (*CXCL9*, *CXCL10*, *CXCL11*, *IFNG* and *TNFA*) were significantly upregulated in antigen-stimulated blood from *M*. *bovis*-infected warthogs, with *CXCL10* showing the greatest upregulation. Therefore, GEAs targeting the *CXCL* genes, show great promise as potential diagnostic tools for detecting *M*. *bovis* infection in suids.

## Materials and Methods

### Animals, sampling and selection of study animals

Heparinized WB, serum and tissue samples were opportunistically obtained from warthogs from bTB endemic areas, as previously described^8^. Tissues were processed for mycobacterial culture and cultures were speciated using genetic region of difference analysis^3,31^. Sera were tested using the Indirect PPD ELISA as previously described^1^. Culture results were used to define warthogs as *M*. *bovis*-infected, whereas the culture and serology results were used to define the uninfected cohort. The Stellenbosch University Animal Care and Use committee provided ethical approval for this study (SU-ACUD15-00029). All methods were performed in accordance with the relevant guidelines and regulations set out in the ethics application.

### Whole blood stimulation and RNA stabilization

The QFT system (Qiagen, Venlo, Netherlands) was used to stimulate warthog WB^8^. Briefly, 1 ml of WB was added to both a Nil tube (QFT-Nil, containing saline) and a TB antigen tube (QFT-TB, containing ESAT-6 and CFP-10 peptides). For optimal antigen exposure, the tubes were repeatedly inverted to ensure thorough mixing and contact of blood with the entire inner surface of the tube. Following incubation for 24 hours at 37 °C, blood was transferred to a 2 ml micro-centrifuge tube and centrifuged at 800 × g for 10 min. Plasma was harvested, and the cell pellet re-suspended in 1.3 ml of RNAlater (Ambion, Austin, TX, USA). Samples were subsequently stored at −80 °C until further analysis.

### RNA extraction and cDNA preparation

The RNAlater-stabilised QFT-TB and QFT-Nil samples of 15 *M*. *bovis*-infected and 17 uninfected warthogs were centrifuged at 15 000 × g for 2 min and the supernatant discarded. The RNA was then extracted from the remaining blood cell pellet using the RiboPure Blood Kit (Ambion) according to the manufacturer’s guidelines. A single modification was made to the elution step; the volume of the elution solution was decreased to 60 µl. A NanoDrop 1000 spectrophotometer (Thermo Fisher Scientific, Wilmington, DE, USA) was used to determine the RNA concentration and quality (260/280 and 260/230 ratio) of each sample. The QuantiTect Reverse Transcription (RT) kit (Qiagen), including a genomic DNA (gDNA) Wipeout step, was used to reverse transcribe 200 ng of RNA from each sample in a final volume of 20 µl, as per manufacturer’s guidelines.

### Sequencing of warthog mRNA transcripts

To obtain warthog mRNA sequences of selected candidate reference (stably expressed in other species) and target genes (used to detect tuberculosis)^9,10,14^, degenerate primers based on the sequences of pig and cow, were designed to anneal to the transcripts of interest. To do this, pig and cow mRNA sequences of these genes were obtained from the Ensembl Genome Browser (http://www.ensembl.org/index.html) and aligned using the Clustal Omega online tool (http://www.ebi.ac.uk/Tools/msa/clustalo/). Using the Primer3Plus online software (http://primer3plus.com/cgi-bin/dev/primer3plus.cgi), primers were designed to anneal to sequences with the greatest identity within the untranslated regions (Table 3). Primers were then used to amplify the entire coding sequence from two randomly selected warthogs using a Veriti 96-Well Thermal Cycler (Applied Biosystems, Foster City, CA, USA). For each transcript, 1 µl of cDNA was added to 12.5 µl of One*Taq* Hot Start 2x Master Mix with Standard Buffer (New England BioLabs Inc., Ipswich, MA, USA), 1 µl of each gene-specific forward and reverse primer (final concentration of 0.5 µM; Integrated DNA Technologies, Coralville, IA, USA) and 9.5 µl nuclease-free water. The reaction was initiated at 94 °C for 15 min, followed by 40 cycles of 94 °C for 30 s, a gene-specific annealing temperature for 30 s (Table 3), and 68 °C for 90 s, and concluded with a final extension at 68 °C for 5 min. The PCR products were sequenced at the Central Analytical Facility (Stellenbosch University, South Africa) using a 3130xl Genetic Analyzer (Applied Biosystems), according to the manufacturer’s guidelines, and analysed using MEGA 7^32^. The warthog mRNA sequences were submitted to the GenBank genetic sequence database (http://www.ncbi.nlm.nih.gov/genbank/) (Table 3). The sequence identity of all warthog derived transcripts were determined by aligning these with those of the pig and cow using the web based multiple sequence alignment tool from EMBL-EB, MUSCLE (https://www.ebi.ac.uk/Tools/msa/muscle/). The result output was calculated as the percent identity.

**Table 3:** Polymerase chain reaction primers and annealing temperatures used to amplify and sequence selected mRNA transcripts of warthogs.

Gene	Forward (5′-3′)	Reverse (5′-3′)	T_annealing_	Accession numbers
*B2M*	ATTCCACCGCCAGCACCGCT	CCCCCTCTACATCTACCTGCT	56 °C	MK333445
*H3F3A*	ATGGCYCGWACMAAGCAGAC	CGRCGWGCYARCTGGATGTC	56 °C	MK333454
*LDHA*	GAAGTGCACTCCCGATTCCT	AGGCTGTCTTAACATTACTGCT	56 °C	MK333450
*PPIA*	TCGTGCTGCCTTGCA	GCTACAGAAGGAATGGTCTG	51 °C	MK333451
*YWHAZ*	RCASAACATCCAGTC	AARTGGTCTACTGTGTAAAT	45 °C	MK333453
*CXCL9*	ACAGRAGTGAYWYYRYYCTACCA	GCCMTCCYYTTYWGGAATTATTTCAG	55 °C	MK333446
*CXCL10*	CAKTSKGAGCCTRCMGCAGAAG	ARTCCAYGGACADTTAGGGCTTSA	57 °C	MK333447
*CXCL11*	TACTCCCTCCAAGAAGAGTATCA	AGCGTTCTTATTTCAGTATTCACAGT	53 °C	MK333448
*IFNG*	TCTGGGCCTGATCGACTGTA	TTTGATCAATGAATCAATATTCCCCA	52 °C	MK333449
*TNFA*	ACYTGARCCCYTCTGAAAA	AAACCAGAAGGRSRTGAG	50 °C	MK333452

The NCBI accession number of each mRNA sequence is listed.

### qPCR design and optimization

Using Primer3Plus and warthog sequences, qPCR primers were designed to span putative exon-exon boundaries for each of the reference and target transcripts. All qPCRs were evaluated at annealing temperatures ranging from 58–62 °C and primer concentrations ranging from 0.25–0.75 µM. Hereafter, optimized reactions were done in triplicate using a CFX96 Touch Real-Time PCR Detection System (Bio-Rad Laboratories Inc., Hercules, CA, USA) and consisted of 5 µl iTaq Universal SYBR Green Supermix (Bio-Rad), 0.5 µl of each gene-specific forward and reverse qPCR primer (at a final primer concentration of 0.5 µM; Integrated DNA Technologies), 1 µl of cDNA and 3 µl of nuclease-free water. The reaction was initiated at 95 °C for 30 s, followed by 40 cycles of 95 °C for 5 s and gene-specific temperatures for 30 s (Table 1), and concluded with a standard melt-curve analysis.

To confirm the specificity of all qPCRs, products from two warthogs were visualized in 1% agarose gel by electrophoresis and sequenced as described above (Table 1). The melt-curve for each qPCR product was characterized and used to confirm qPCR specificity of subsequent reactions. To confirm the absence of non-specific amplification, all qPCRs had a no-template control. In order to show the absence of amplifiable gDNA in RNA, qPCRs were performed with four randomly selected samples that had been treated with gDNA Wipeout buffer, but had not been reverse transcribed. For each qPCR, quantification cycles (C_q_) were automatically determined by the CFX Manager Software (Bio-Rad). The efficiency of the qPCRs were determined by analysing a serial dilution (64 fold) of a pooled cDNA sample for each gene^33^. To validate the use of the relative quantification method, amplification efficiencies of the reference and target genes were compared as previously described^34^.

### Data analysis

To determine the intra-assay variability of each qPCR, the coefficient of variance was calculated for triplicate reactions. The relative expression stability of the reference genes were compared by analysing qPCR Cq values for QFT-Nil and QFT-TB samples of four randomly selected *M*. *bovis*-infected warthogs, using the geNorm applet in Microsoft Excel^35^ and the NormFinder Excel Add-In^36^. The most stable reference gene was selected and used to further analyse the relative gene expression of the target genes.

The relative expression of each target gene was normalised by subtracting the Cq value of the selected reference gene from the Cq value of the target gene. This was done to calculate the relative abundance of the target gene mRNA for each sample (i.e. ΔCq). Thereafter, the ΔCq value derived from the QFT-Nil sample was subtracted from the ΔCq value derived from the QFT-TB sample for all animals (i.e. ΔΔCq). The relative fold change (2^−ΔΔCq^) was used to derive a QFT GEA result as a measure of the upregulation of the target transcript in response to antigen stimulation^34^.

The assay results for all target genes were then analysed in GraphPad Prism version 5 (GraphPad Software Inc., La Jolla, CA, USA). A Mann-Whitney U test was used to determine if there was a significant difference in the antigen-induced expression (2^−ΔΔCq^ value) of each gene between *M*. *bovis*-infected and uninfected warthogs. Receiver operator characteristic (ROC) curve analysis was used to determine cut-off values for each target gene by using Youden’s index^37^. Results with a p-value < 0.05 were considered statistically significant.

## Supplementary information

Supplementary information
