# Supplementary material for: Cytokine gene expression assay as a diagnostic tool for detection of Mycobacterium bovis infection in warthogs (Phacochoerus africanus)
Source: Sci Rep. 2019 Nov 11;9:16525. doi: 10.1038/s41598-019-53045-0 (PMC6848127; doi:10.1038/s41598-019-53045-0)
Supplement: Supplementary file 1 — Supplementary information [file 41598_2019_53045_MOESM1_ESM.pdf]

Cytokine gene expression assay as a diagnostic tool for detection of *Mycobacterium bovis* infection in warthogs (*Phacochoerus africanus*)

Eduard O. Roos[a*], Leeré A. Scott[a], Sedzani Ndou[a], Francisco Olea-Popelka[b], Peter E. Buss[c], Lin-Mari de Klerk-Lorist[d], Robin M. Warren[a], Paul D. van Helden[a], Tashnica T. Sylvester[a], Michele A. Miller[a], Sven D.C. Parsons[a]

[a] Department of Science and Technology/National Research Foundation Centre of Excellence for Biomedical Tuberculosis Research, South African Medical Research Council Centre for Tuberculosis Research, Division of Molecular Biology and Human Genetics, Faculty of Medicine and Health Sciences, Stellenbosch University, PO Box 241, Cape Town 8000, South Africa

[b] Department of Clinical Sciences, College of Veterinary Medicine and Biomedical Sciences, Colorado State University, 300 W. Drake Rd, Fort Collins, Colorado 80523, USA

[c]Veterinary Wildlife Services, South African National Parks, Kruger National Park, Private Bag X402, Skukuza, 1350, South Africa

[d]Office of the State Veterinarian, Kruger National Park, PO Box 12, Skukuza 1350, Department of Agriculture, Forestry and Fisheries, South Africa

[*]Present address: The Pirbright Institute, Ash Rd, Pirbright, Woking GU24 0NF, United Kingdom

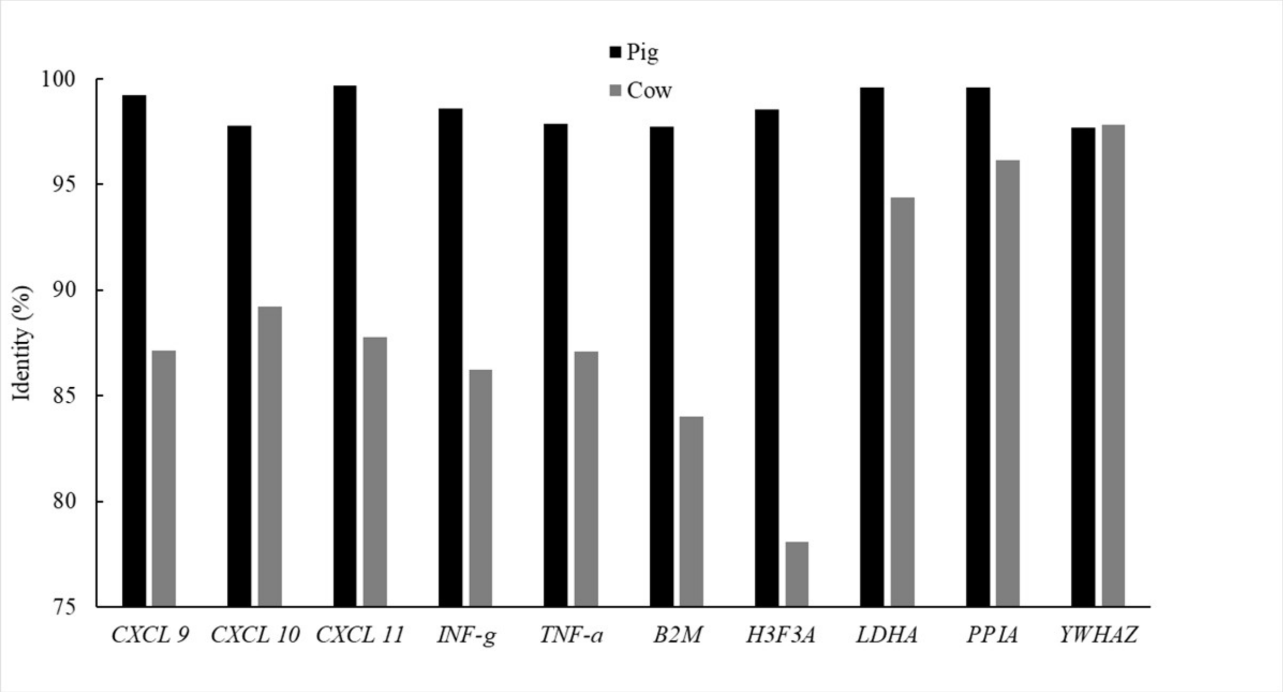

Figure S1 mRNA gene transcript identity of the warthog with that of the domestic pig and domestic cow. The warthog transcripts were most like that of the domestic pig, with all transcripts having an identity of more than 97%.

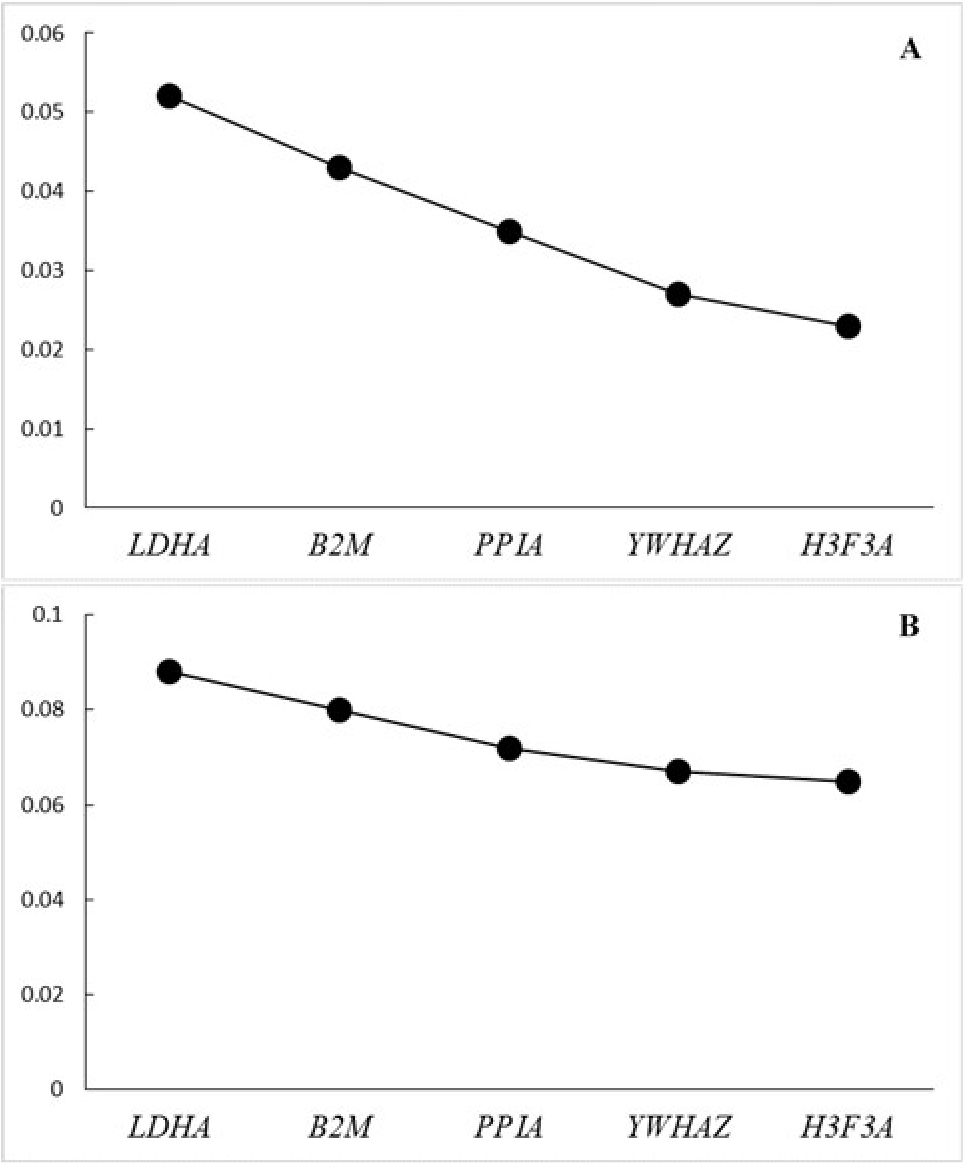

Figure S2 Gene expression stability of selected reference genes in whole blood, from four randomly selected warthogs, incubated in QuantiFeron TB Antigen and Nil tubes. Stability was calculated using (A) NormFinder Excel Add-In and (B) geNorm Excel applet.

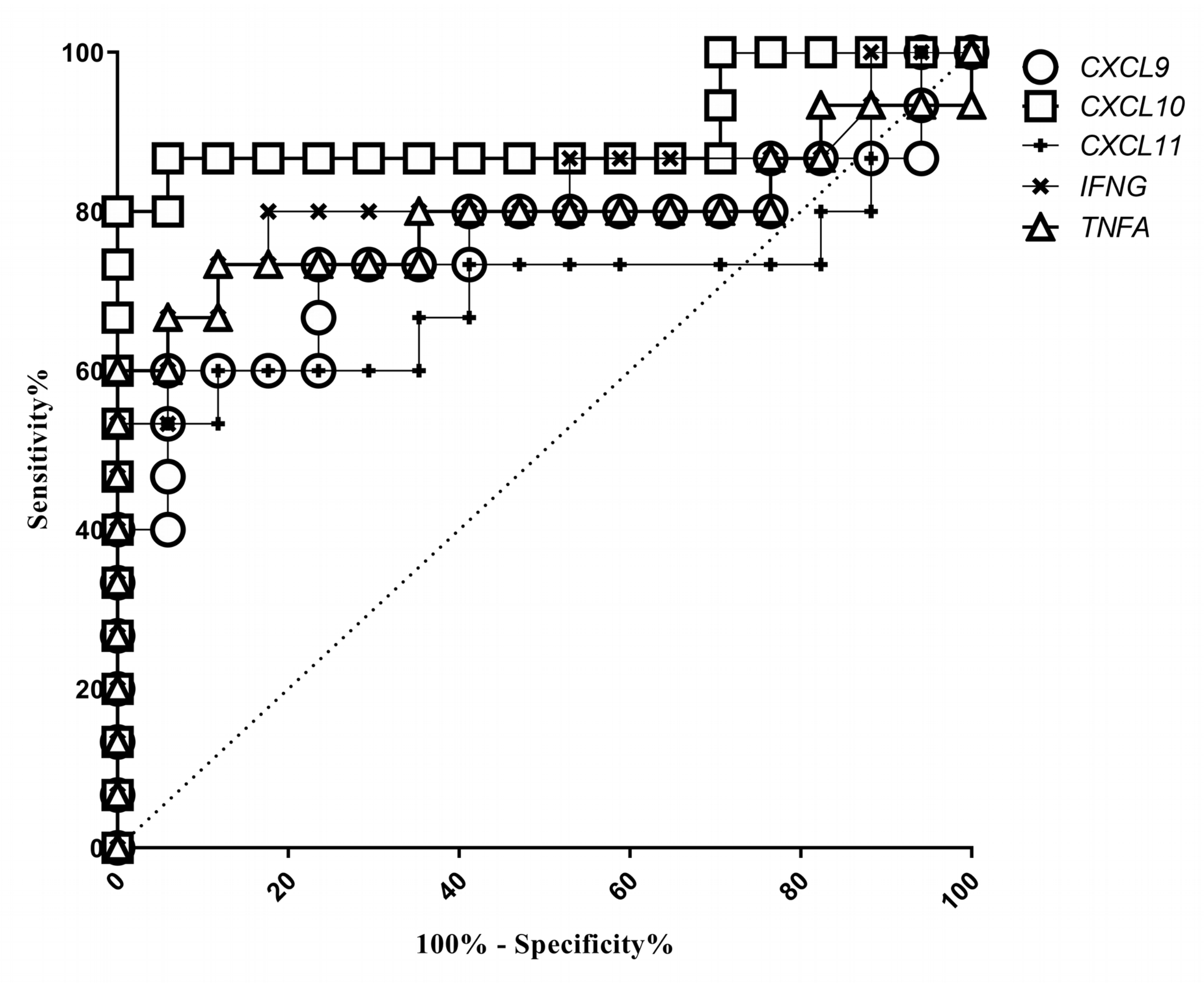

Figure S3 ROC curve analysis for each target gene based on *M. bovis* culture results. The dotted line represents the line of no-discrimination and AUC ranged from 0.71-0.90.
